# Satellite Based Assessment of Hydroclimatic Conditions Related to Cholera in Zimbabwe

**DOI:** 10.1371/journal.pone.0137828

**Published:** 2015-09-29

**Authors:** Antarpreet Jutla, Haidar Aldaach, Hannah Billian, Ali Akanda, Anwar Huq, Rita Colwell

**Affiliations:** 1 Department of Civil and Environmental Engineering, West Virginia University, Morgantown, WV, United States of America; 2 Department of Civil and Environmental Engineering, University of Rhode Island, Kingston, RI, United States of America; 3 Maryland Pathogen Research Institute, University of Maryland, College Park, MD, United States of America; 4 Centre for Bioinformatics and Computational Biology, University of Maryland, College Park, Maryland, United States of America; 5 Bloomberg School of Public Health, Johns Hopkins University, Baltimore, Maryland, United States of America; University California Los Angeles, UNITED STATES

## Abstract

**Introduction:**

Cholera, an infectious diarrheal disease, has been shown to be associated with large scale hydroclimatic processes. The sudden and sporadic occurrence of epidemic cholera is linked with high mortality rates, in part, due to uncertainty in timing and location of outbreaks. Improved understanding of the relationship between pathogenic abundance and climatic processes allows prediction of disease outbreak to be an achievable goal. In this study, we show association of large scale hydroclimatic processes with the cholera epidemic in Zimbabwe reported to have begun in Chitungwiza, a city in Mashonaland East province, in August, 2008.

**Principal Findings:**

Climatic factors in the region were found to be associated with triggering cholera outbreak and are shown to be related to anomalies of temperature and precipitation, validating the hypothesis that poor conditions of sanitation, coupled with elevated temperatures, and followed by heavy rainfall can initiate outbreaks of cholera. Spatial estimation by satellite of precipitation and global gridded air temperature captured sensitivities in hydroclimatic conditions that permitted identification of the location in the region where the disease outbreak began.

**Discussion:**

Satellite derived hydroclimatic processes can be used to capture environmental conditions related to epidemic cholera, as occurred in Zimbabwe, thereby providing an early warning system. Since cholera cannot be eradicated because the causative agent, *Vibrio cholerae*, is autochthonous to the aquatic environment, prediction of conditions favorable for its growth and estimation of risks of triggering the disease in a given population can be used to alert responders, potentially decreasing infection and saving lives.

## Introduction

Cholera, a life-threatening diarrheal disease, has been shown to be transmitted by drinking water contaminated with the causative agent, *Vibrio cholerae*, and the disease remains a global public health threat. Recent studies have established that cholera occurs in three forms: epidemic (a sudden outbreak in a previously relatively disease-free region and primarily inland), endemic (recurring essentially annually and predominantly along coastal regions) [1], and mixed-mode (a combination of epidemic and endemic disease occurrence). Epidemic and endemic cholera show significantly different mortality rates, with epidemic cholera having higher mortality, over 6% in some cases. The mortality rates reported for recent epidemics include Haiti 6.4% in 2010, Madagascar 6% in 2000, Zimbabwe 4.3% in 2008–09, and Nigeria 3.8% in 2010 [2]. Current methods are effective in treating cholera, e.g., oral rehydration, intravenous saline solution, and vaccines; but providing safe water access remains the most effective method to prevent outbreaks. *V*. *cholerae* exists naturally in the aquatic environment. Hence growth of the bacterium is strongly influenced by environmental conditions. Furthermore, *V*. *cholerae* is reemerging in many areas in the form of newly virulent serotypes, such as *V*. *cholerae* 0139, complicating traditional treatments. Because it is not realistic or feasible to consider eradication of the pathogen from the aquatic environment, a fundamentally transformational approach is needed to design mitigation strategies to protect against the disease. If prediction of a probable cholera epidemic using large scale hydroclimatic data is possible, appropriate resources and treatment can be dedicated to a given location in advance.

The majority of cholera research over the last several decades has focused on endemic cholera regions, primarily Bangladesh, where temporal disease surveillance data have been accumulated. Research on *V*. *cholerae*, the agent itself, also is extensive, both on its micro-environment (biological processes associated with the bacteria) and macro-environment (large scale hydrological, ecological, and climatological processes related to outbreaks of the disease). Both sets of information are crucial to understand how the disease is triggered in a specific geographic region and how it can spread in that population.

Association of climate variables with cholera in Africa is relatively unexplored and only a few studies have attempted to understand quantitatively the disease dynamics to predict cholera outbreaks. Africa, in general, is considered to be a new homeland of cholera [3]. In 2006, Africa reported 234,349 cases of cholera to the World Health Organization (WHO), accounting for 99% of the officially notified global cholera [3]. Two years of cholera outbreak data from KwaZulu-Natal in South Africa were shown to be statistically associated with sea surface temperature (SST), precipitation, and coastal phytoplankton, the latter being the surrogate indicator of zooplankton, the natural host of the cholera vibrio [4], as had been demonstrated earlier for the Bay of Bengal [5]. However, the studies were carried out where cholera is endemic. The impact of temperature and rainfall, both associated with climate change, on cholera was studied in Tanzania and the conclusion was that temperature is significantly associated with cholera, i.e., a one degree Celsius increase in air temperature increases relative risk of cholera by 15–29% [6]. A positive correlation of historical precipitation and temperature data with hospital records of disease for children under the age of thirteen was observed in South Africa [7]. Diarrhea (although not specifically attributed to cholera) was one of the diseases considered, comprising 42.4% of the reported hospital cases. Regression analysis showed the combined effect of temperature and rainfall explained approximately 38% of disease occurrence, including other diseases as well as diarrhea [7]. A predictive cholera study in Africa examined diarrheal incidence in Botswana over a 30-year period, in relation to several climatic variables, including rainfall, minimum temperature, and vapor pressure [8]. In that study, diarrheal diseases showed annual bimodal occurrence, predictable by a one-month lag in the climatic variables [8]. Other studies, although not predictive in design, linked climatic variables, e.g., precipitation, temperature, and humidity, to outbreaks of cholera, based on historical data [9–11]. Most of the studies were strictly correlational, lacking a physical hypothesis with respect to cholera and large scale geophysical variables. Relevant is an observation made in a review [12], where it was qualitatively concluded that precipitation may be a key variable for understanding modalities of seasonal variability in the occurrence of disease in human populations. Recently, a hypothesis was proposed by Jutla et al., [1] concerning large scale hydroclimatic control of cholera outbreaks occurring inland away from the coasts and in epidemic form, concluding that elevated temperatures create environmental conditions favorable for growth of the cholera bacteria. When followed by above average rainfall and appropriate transmission mechanisms (i.e. ineffective or catastrophic destruction of sanitation infrastructure resulting in open flow of domestic wastewater), a cholera epidemic can be expected [1]. The hypothesis was developed from historical data on cholera in South Asia.

The first cases of cholera in Zimbabwe were reported to the World Health Organization in August, 2008 [13]. Between August, 2008, and June, 2009, a total of 98,522 cholera cases and 4,282 deaths were reported [14]. The case fatality ratio (CFR), i.e., the ratio of deaths to total cases reported, peaked in January, 2009 [14]. CFRs for the entire Zimbabwe epidemic were much higher than in those areas of the country where appropriate treatment was available, being highest in the rural areas of Zimbabwe, away from the treatment centers [14]. Based on the information available, the cholera outbreak originated in Chitungwiza, a subsection of the capital city of Harare, in the province of Mashonaland East [15]. There are no published results documenting the role of environmental factors associated with cholera outbreaks in Zimbabwe. Large-scale hydroclimatic processes can be estimated using remote sensing and linked with epidemiological data to assess risk of disease occurrence [16]. These technologies provide measurements for geophysical variables at varying spatial and temporal scales [17]. Employing the epidemic cholera hypothesis, we established the objective of this study to underscore sensitivity in satellite derived precipitation and air temperature data and to determine their association with timing and spatial variability of disease outbreaks in Zimbabwe, thereby establishing hydroclimatic predictability of the occurrence of cholera.

**Fig 1:**
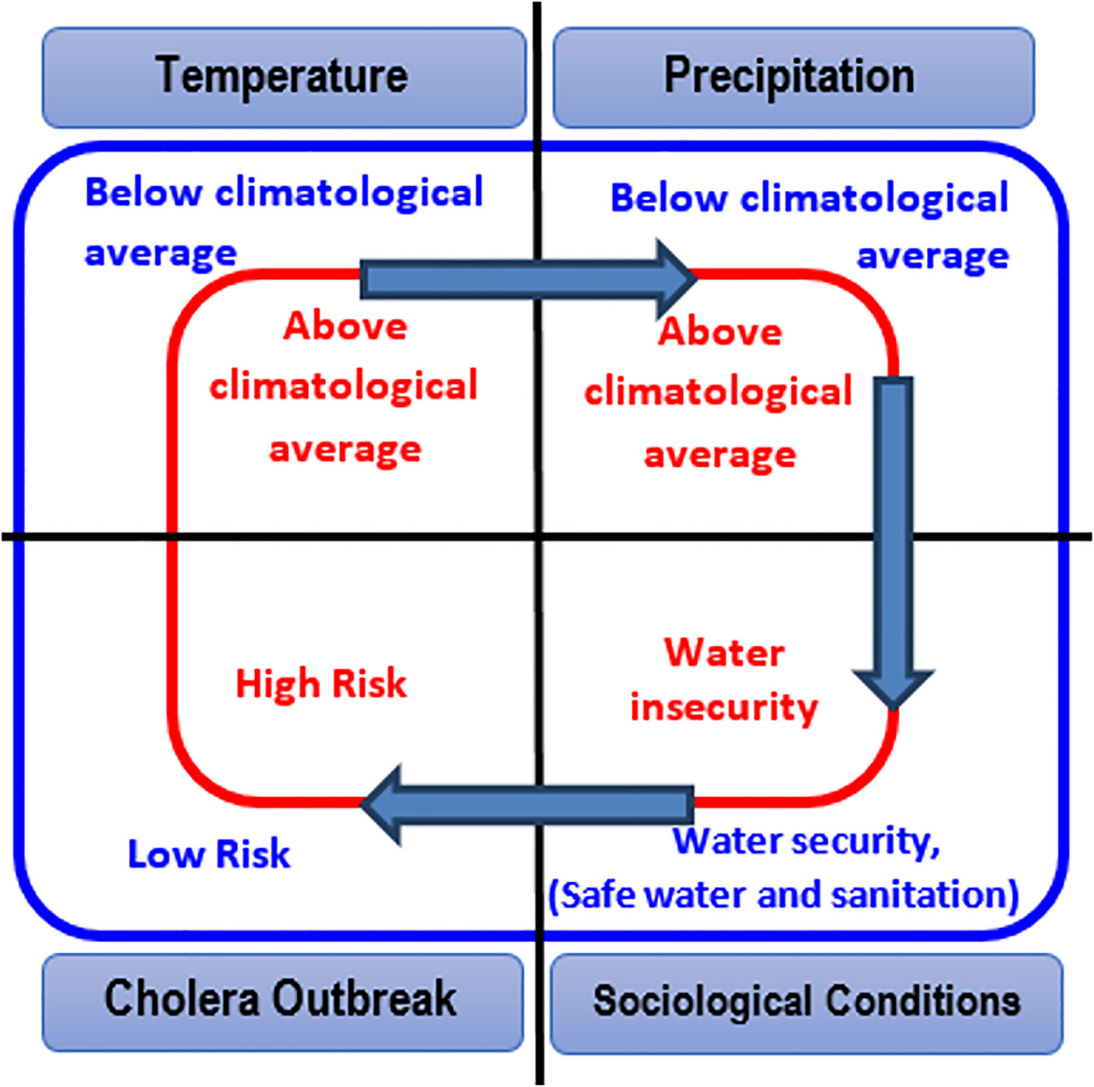
Theoretical hypothesis linking hydroclimatic conditions with sociological aspects and epidemic cholera.

Our previous study [1] developed the theoretical aspects (Fig 1) of the working hypothesis- that warm temperatures, followed by heavy precipitation and in combination with societal factors related to water insecurity (such as natural disasters, political instability) will lead to conditions where interaction of a population with unsafe water will increase, and result in an epidemic of cholera. Within this context, temperature and precipitation serve as the large scale climatic processes that aid in growth and proliferation of cholera bacteria in the aquatic environment [18,19]. In the absence of adverse societal conditions, the chance of occurrence of cholera diminishes. However, bacteria in the environment remain viable agents of disease. The present study tests this hypothesis using data from Zimbabwe where a major cholera epidemic was reported in 2008. To provide supportive evidence, the hypothesis was validated using data for five additional regions of Africa where cholera was reported during the past few years. The challenge of epidemic cholera is that it occurs sporadically. Hence detailed time series are generally not available, limiting determination of the role of hydroclimatic processes in disease outbreaks. In this study, historical time series data on diarrhea prevalence collected from the Indus River basin (details in Materials and Methods section) were used to support (refute) the hypothesis. A compartmental mechanistic model was calibrated and validated to understand the dynamics of interaction between cholera and climatic processes for Zimbabwe. Key innovative aspects of this study are spatial estimation of precipitation by satellite and global gridded air temperature, capturing sensitivities in hydroclimatic conditions that permitted identification of the location in the region where the disease outbreak began.

## Materials and Methods

### Epidemiological data

The climate of Zimbabwe, located in sub-Saharan Africa, is tropical and its rainy season occurs from October through March. Cholera data for Zimbabwe were obtained from the Zimbabwe Ministry of Health and Child Welfare (weekly scales from November 13, 2008 to July 31, 2009), for the Mashonaland East region (details of the data are available from Mukandavire et al. [20]). Historical hydroclimatological data for the Indus River basin were assembled by collecting and processing historical climatological data in the annual reports of the Meteorological Reporter to the Government of India, covering1875 to 1900. Cholera mortality data for the Indus River basin were obtained from statistical statements appended to the Annual Reports of the Sanitary Commissioner to the Government of Punjab, 1875–1900 (Jutla et al. [1]).

### Climate data

Daily precipitation data collected by satellite (Tropical Rainfall Measuring Mission (TRMM) included monthly data product 3B43 at 0.25x0.25 degree resolution) obtained for 1998 to 2013. Air temperature data were obtained from the Global Historical Climatology Network version 2 Climate Anomaly Monitoring System (GCHN CAMS-0.5x0.5 degree resolution) for the same time period(bounding coordinates 15°S to 23°S latitude and 25°E to 33.5°E longitude). Precipitation and air temperature were linked with the cholera epidemics that occurred during the time period of this study.

### Spatial data analysis

The study was designed to determine hydroclimatic conditions triggering the 2008 cholera outbreak in Zimbabwe. To apply the epidemic cholera hypothesis of Jutla et al. [1], calculation of monthly anomalies (subtraction of month value from the average of 15 years of monthly data) for precipitation and temperature was done. These were calculated for each pixel in both datasets, e.g., daily precipitation data obtained from TRMM sensors were converted to monthly scales for fourteen years for all pixels covering Zimbabwe. An average for 15 years was calculated and then subtracted from each month, resulting in a positive or negative value (thereafter referred to as anomaly). A positive anomaly implied that month received greater precipitation from average over the given pixel in a fifteen year period. An additional step of analysis was determination of percent deviation each month from the average for that month, using the following equation:

$$Deviation_{month,year}\left(\%\right)=\left[\frac{Month\;value{\;}_{Precipitation\;/Temperature}-Average\;value{\;}_{Precipitation\;/\;Temperature}}{Average\;value{\;}_{Precipitation\;/\;Temperature}}\right]\times 100$$

For example, *% Deviation* for July, 2008 was calculated by subtracting precipitation during the month from the 15 year average precipitation for July and dividing by the 15 year average precipitation for July. Air temperature data were similarly processed, with the exception that monthly temperature data were employed. Student t-test (two tail) was used to determine if a probability value of less than 0.05 (95% confidence level) rejected the null hypothesis of zero. In order to overcome limitation of data availability, analysis of recent outbreaks of cholera in five other parts of Africa was done. Table 1 provides a summary for the five regions, including months in which cholera outbreaks [21–31] had been reported.

**Table 1:** Recent cholera epidemics in the African continent.

Country	Region(Case Fatality Ratio)	Outbreak Month	Coordinates for data	Tprob^a^(Two month lead)	Pprob^b^(One month lead)	Societal disturbance	Source
**South Sudan**	Juba (5%)	April 2014	4°-5°N; 31°-32°E	0.021(Feb 14)	0.005(March 14)	Civil unrest	[21,22]
**Central African Republic (CAR)**	Mongoumba(13%)	September2011	4°-5°N; 18°-19°E	<0.001(July 11)	0.101(August 11)	Natural Hazard (Floods)	[23,24]
**Rwanda**	Rutsiro(50%)	November2012	2°-1°S;29.5°-30.5°E	<0.001(Sept 12)	<0.001(October 12)	Natural Hazard(Floods)	[25,26]
**Cameroon**	Nord Region (13%)	September2009	9.5°-10.5°N; 14°-15°E	0.012(July 09)	0.023(August 09)	Natural Hazard(Floods)	[27–29]
**Mozambique**	Delgado(1%)	February2013	12.5°S-11.5°S;39°-40°E	<0.001(Dec 12)	0.002(January 13)	Natural Hazard(Floods)	[30,31]

^a^Tprob, for temperature, is the probability (according to two tail t-test) that value in the month is equal to zero (difference between value for that month and the long term average should be zero). For example, a probability value of less than 0.05 (95% confidence level) rejects the null hypothesis that the value is equal to zero.

^b^Pprob is calculated similarly to Tprob, but for precipitation. Coordinates indicate regions where TRMM data were obtained

### Logistical regression analysis

Binomial logistical regression models were developed to estimate the probabilistic likelihood of cholera in the Indus River Basin. Output from the statistical software package MINITAB was compared, using the Goodman-Kruskal Gamma measure of association [32] and the Hosmer-Lemeshow test for goodness of fit [33]. The Hosmer-Lemeshow test was conducted by subgrouping probabilities as deciles of fitted values obtained on monthly scales. The measure of association establishes relationships between predicted probabilities of response variable (above average cholera incidence) and predictors (air temperature and precipitation). Goodness of fit using Goodman-Kruskal Gamma is a non-parametric rank correlation and ranges between -1 to 1 with 1, representing perfect association.

### Mechanistic disease model

Since epidemic cholera is sporadic by definition [1], therefore the long-term time series was not available for Zimbabwe. A mechanistic population based compartmental model, Susceptible-Exposed-Infected-Recovered (SEIR), was developed to analyze and supplement the empirical observations. Details of model development (Fig A and Table A in S1 File), calibration and parameters (Table B in S1 File) are appended in the supplementary information (S1 File).

## Results and Discussion

### Spatial data analysis for Zimbabwe

**Fig 2:**
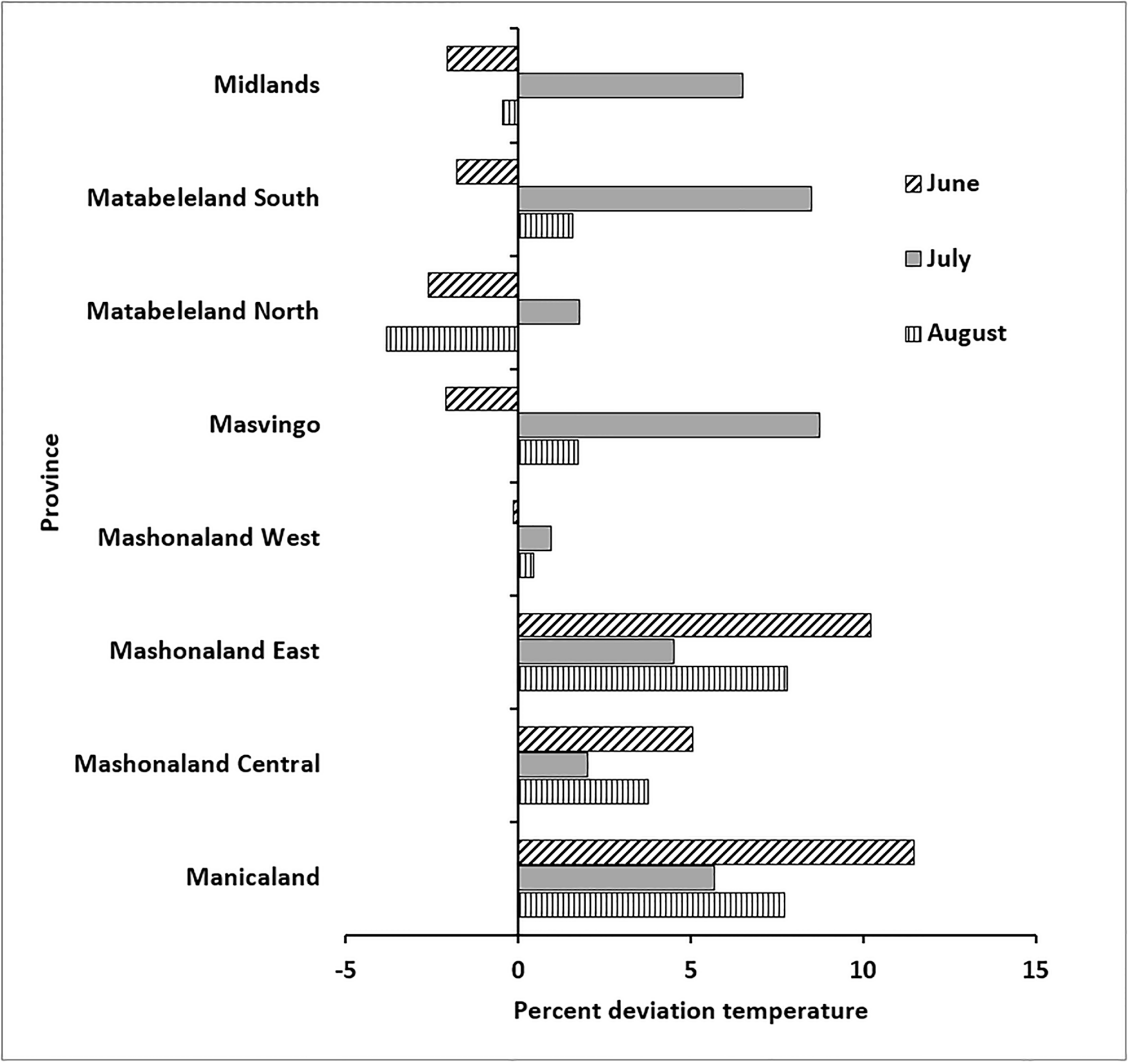
Change in air temperature for June, July and August 2008. A positive value represents temperature higher than the 15-year average; a negative value represents temperature lower than the 15-year average.

**Fig 3:**
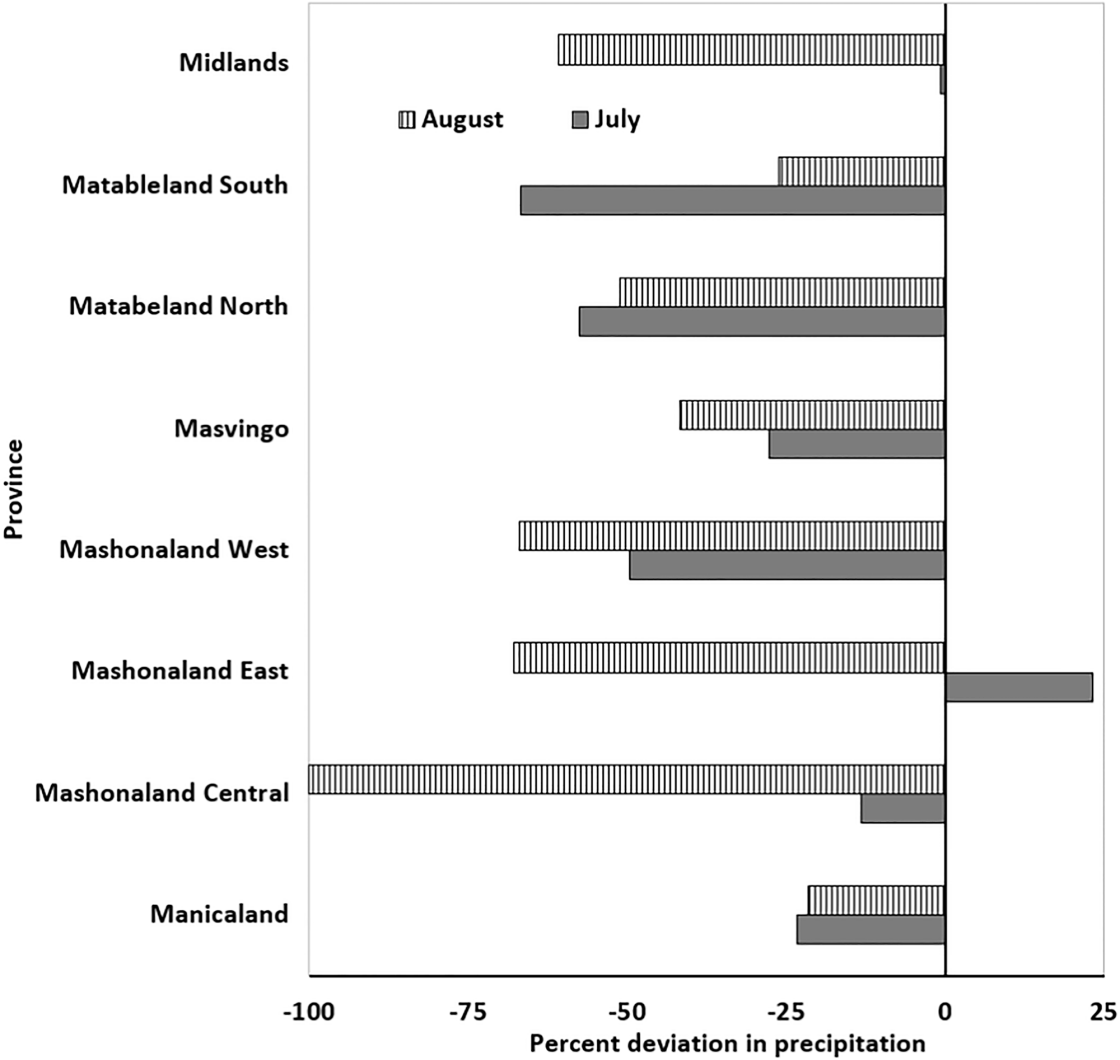
Change in precipitation for July and August 2008. A positive value represents rainfall higher than the 15-year average; a negative value represents rainfall lower than the 15-year average.

The cholera outbreak in Zimbabwe began in Chitungwiza, a city in the Mashonaland East province in August, 2008. Hydroclimatic processes for the months of June through August are critical, since departure from normal for the Mashonaland East province can provide conditions favorable for growth of cholera bacteria in the aquatic environment. It must be noted that the precise location of the region of a disease outbreak cannot be determined because of limitations in the resolution of satellite data. However, provincial analysis (average of all pixels in a particular province) showed air temperature in Mashonaland (East and Central), as well as in Manicaland, were ca. 2.0 to 12.0% higher than average (Fig 2). If anomalous temperatures in a region are followed by heavy precipitation, the risk of a cholera outbreak increases. Fig 3 shows precipitation in Mashonaland East had a positive anomaly during the month of July, 2008, whereas the rest of Zimbabwe experienced less than normal precipitation during July and August, 2008. Precipitation was lower than the climatic average, based on the data available for two months before the outbreak, except in the province of Mashonaland East, where rainfall was ca. 25% higher than average. Results are not shown, but data for the preceding months indicate it was also below the 15-year average. Elevated temperature, followed by heavy precipitation have been found to be indicators of conditions optimum for cholera, notably where the drinking water source and sanitation infrastructure are poorly maintained or unavailable.

**Fig 4:**
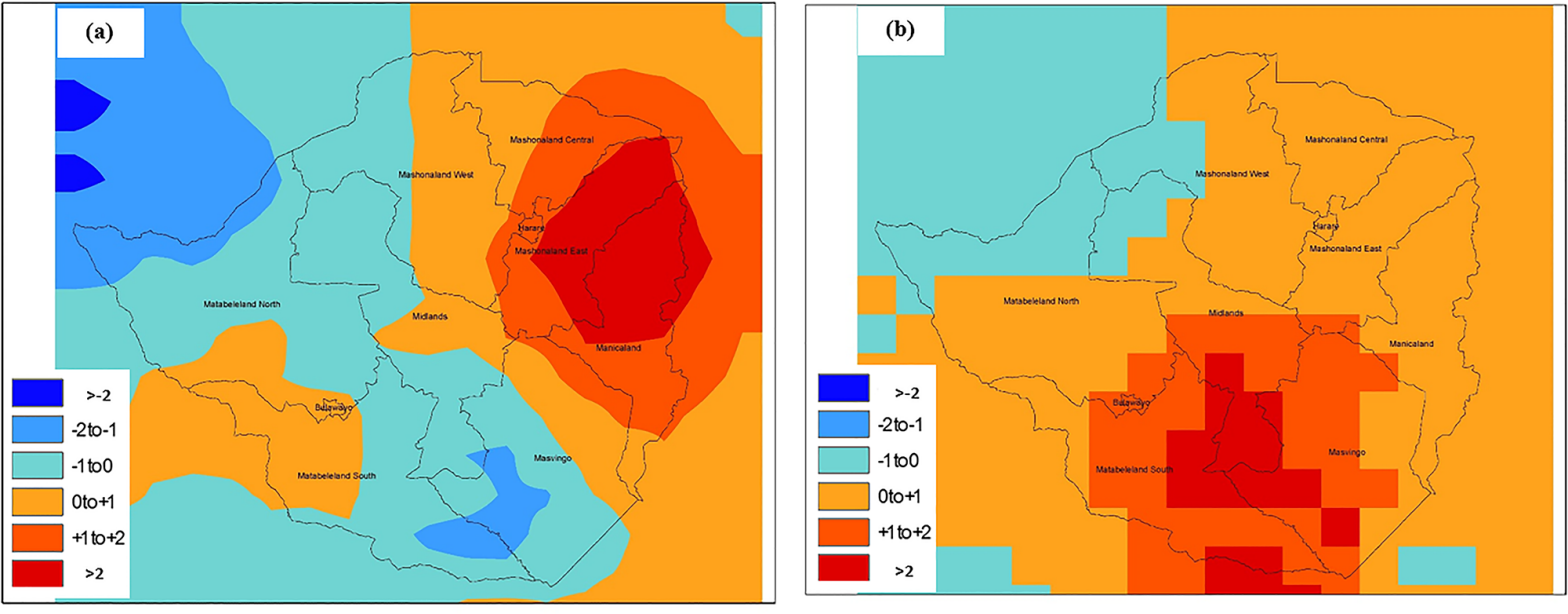
Spatial temperature change from 15-year Average for (a) June and (b) July 2008.

**Fig 5:**
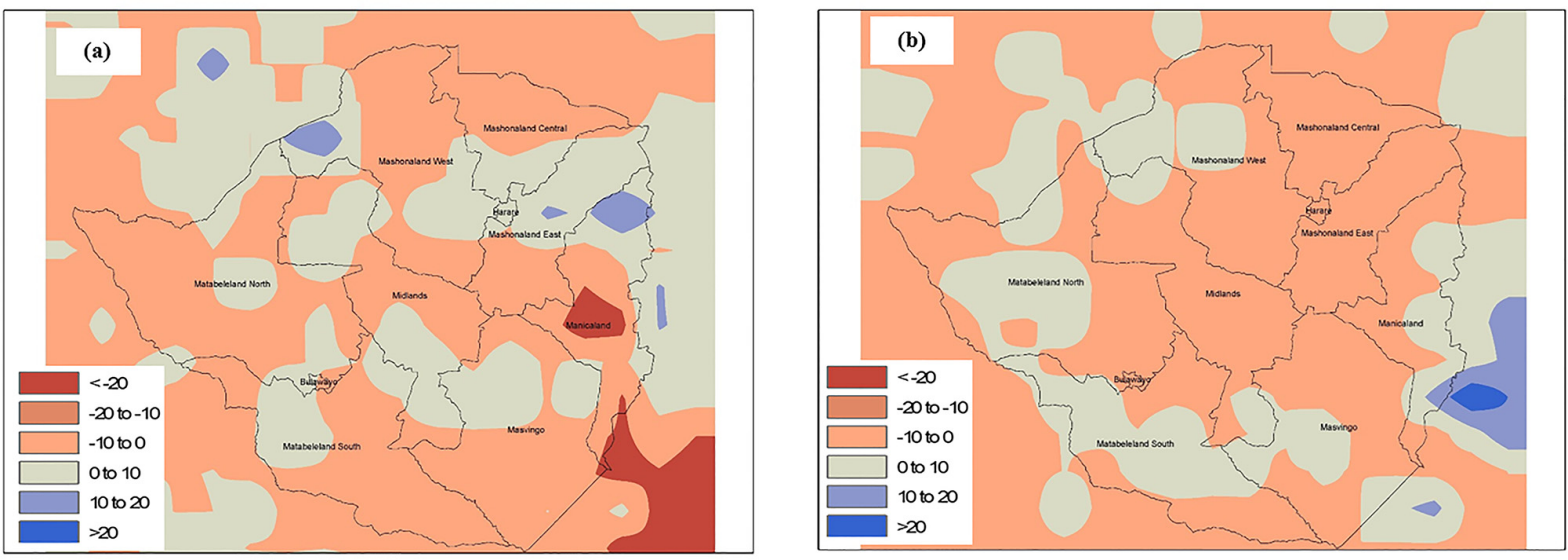
Spatial precipitation change from 15-year Average for (a) July and (b) August 2008.

Supportive evidence was obtained when spatial anomaly maps of temperature and precipitation were plotted, with expectation that if conclusions from the provincial analysis were valid, then differentiation in the patterns of hydroclimatic variability would be observed at spatial scales. Fig 4 shows interpolated air temperature anomaly maps for the entire country of Zimbabwe for June, July, and August, 2008. It can be observed that Mashonaland (East, Central), as well as Manicaland, had temperature anomalies between +1 to +3% (changes greater than 2% were statistically significant at 95% confidence interval using two tail t-tests) during June, 2008, and a positive departure from the average during July, 2008. That is, warm conditions were present that were suitable for growth of the cholera bacteria in the environmental water system, mainly the Nyatsime and Manyame rivers. Following the period of elevated temperature, Mashonaland East had heavier than average precipitation, up to 10% (Fig 5a) during July, 2008.

Parts of Matabeleland South and Masvingo provinces (Fig 4) also experienced anomalous temperatures and greater precipitation during August, 2008. Therefore, these regions should have experienced cholera in September, but disease outbreak occur if favorable environmental conditions are accompanied by impaired water and sanitation infrastructure. While the Mashonaland East region experienced mixing of sewage contaminated river water with drinking water in both Chitungwiza and Harare cities [34], none of the regions in Matabeleland South and Masvingo provinces reported such incidents, but the disease subsequently spread throughout the country via secondary (human to environmental) transmission.

**Fig 6:**
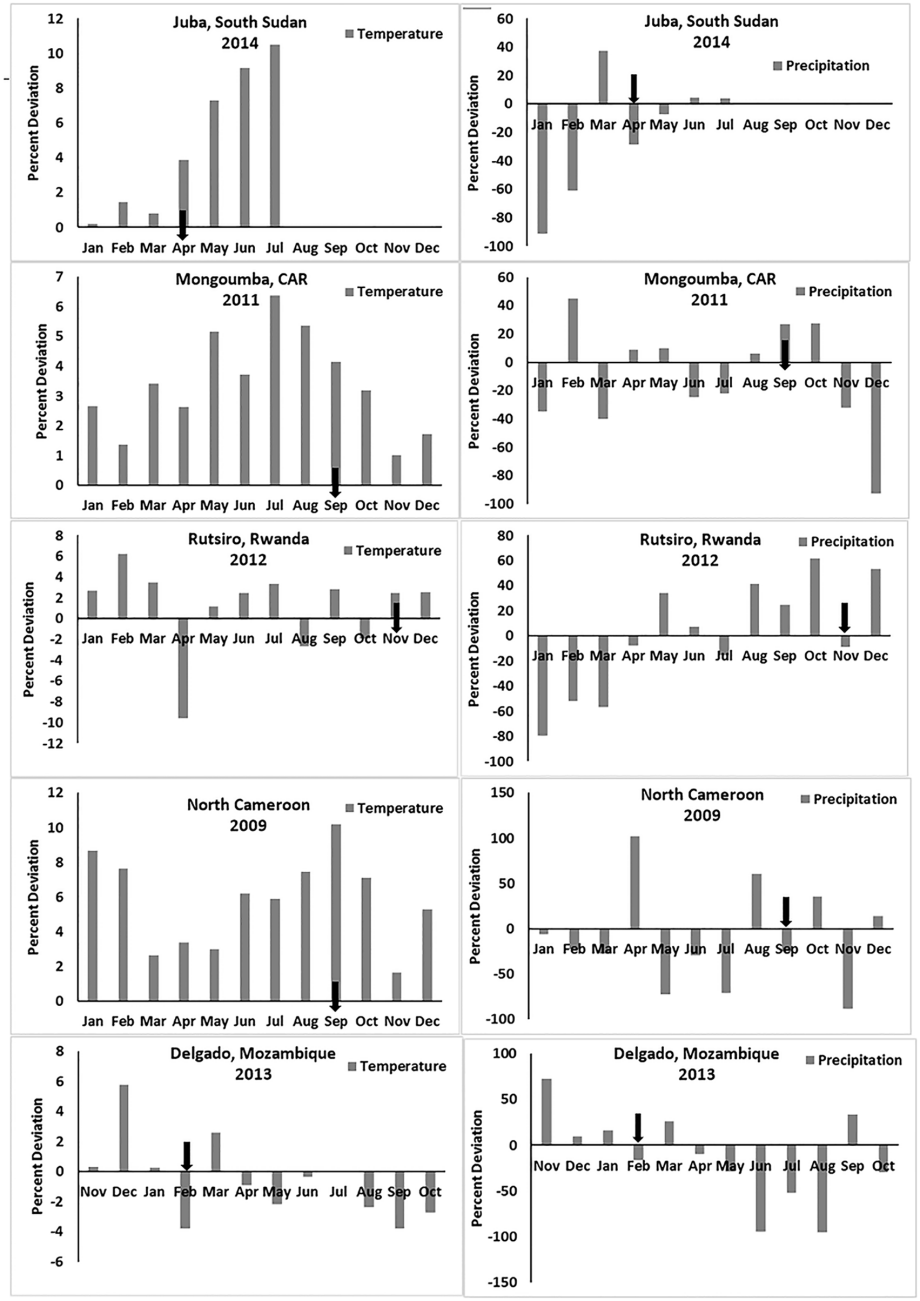
Precipitation and temperature anomalies for five regions in Africa. Black arrows on the x-axis of plots indicates month when cholera was first reported.

If empirical observations for the 2008 Zimbabwe cholera are valid, similar trends should be observed in other regions. Table 1 provides a summary for five regions that includes those months for which first cholera cases were reported. Fig 5 was constructed similarly as for Fig 2, but includes all of the five other regions. The data show that anomalous above average air temperatures, followed by above average precipitation, occurred at all sites, and at least one month in advance. Fig 6 shows the month when cholera was first reported for that region, and the corresponding analysis presented in Table 1 indicates that the observed anomalies of precipitation and temperature were statistically significant at 90% confidence intervals (using two-tail t-test), corroborating the hypothesis for cholera in Zimbabwe in 2008.

### Analysis of historical epidemic cholera for the Indus River Basin

**Fig 7:**
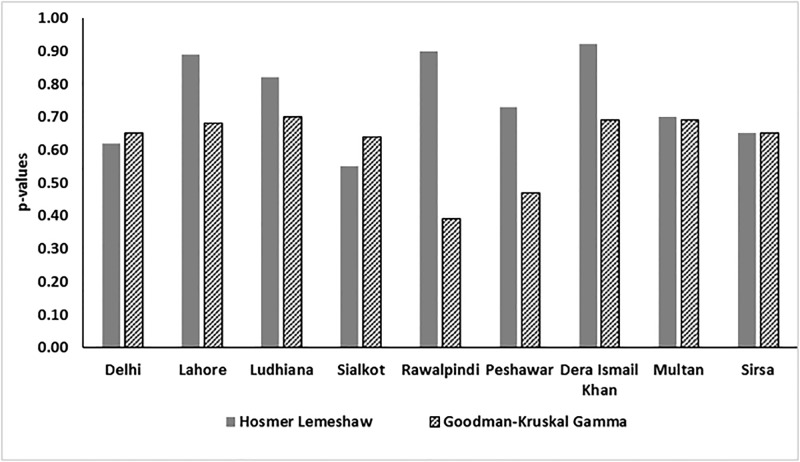
Statistical metrics for measure of association of cholera with air temperature and precipitation in the Indus River Basin. (Somers’D and Goodman Kruskal Gamma p-values were multiplied with 100 for comparison purposes).

To validate the findings and determine whether the pattern of air temperature and precipitation would be observed elsewhere, we employed the binomial logistical regression models to analyze 25 years of historical cholera data for the Indus River basin region (Table C in S1 File). The model estimates probability of occurrence of cholera for nine locations in the Indus River Basin (Delhi, Lahore, Ludhiana, Sialkot, Rawalpindi, Peshawar, Dera Ismail Khan, Multan, and Sirsa). Additional details for these locations have been published (Jutla et al., 2013). Fig 7 shows results of performance of the model with respect to metrics for the association between predictor (cholera) and predictand (two month lead in air temperature and one month lead in precipitation). If the hypothesis for Zimbabwe cholera is valid, statistically significant values for model performance indicators should be observed for Indus River historical cholera. If p-values for the Hosmer-Lemeshow calculations are 0.52 to 0.85, the indication is insufficient evidence to claim the model does not fit the data. The p-values for Hosmer-lemeshow tests calculated in this instance were greater than 0.05 (statistical confidence at 95%), hence does not reject the null hypothesis that the model does not adequately fit the data. Similarly, the measure of association ranged between 0.35 to 0.70, suggesting a limited predictive capability of the model. The results statistically support the hypothesis of role for hydroclimatic conditions in epidemic cholera, with the caveat that only limited information is available concerning conditions of water and sanitation infrastructure during the study time period in the Indus River Basin.

### Mechanistic model results

**Fig 8:**
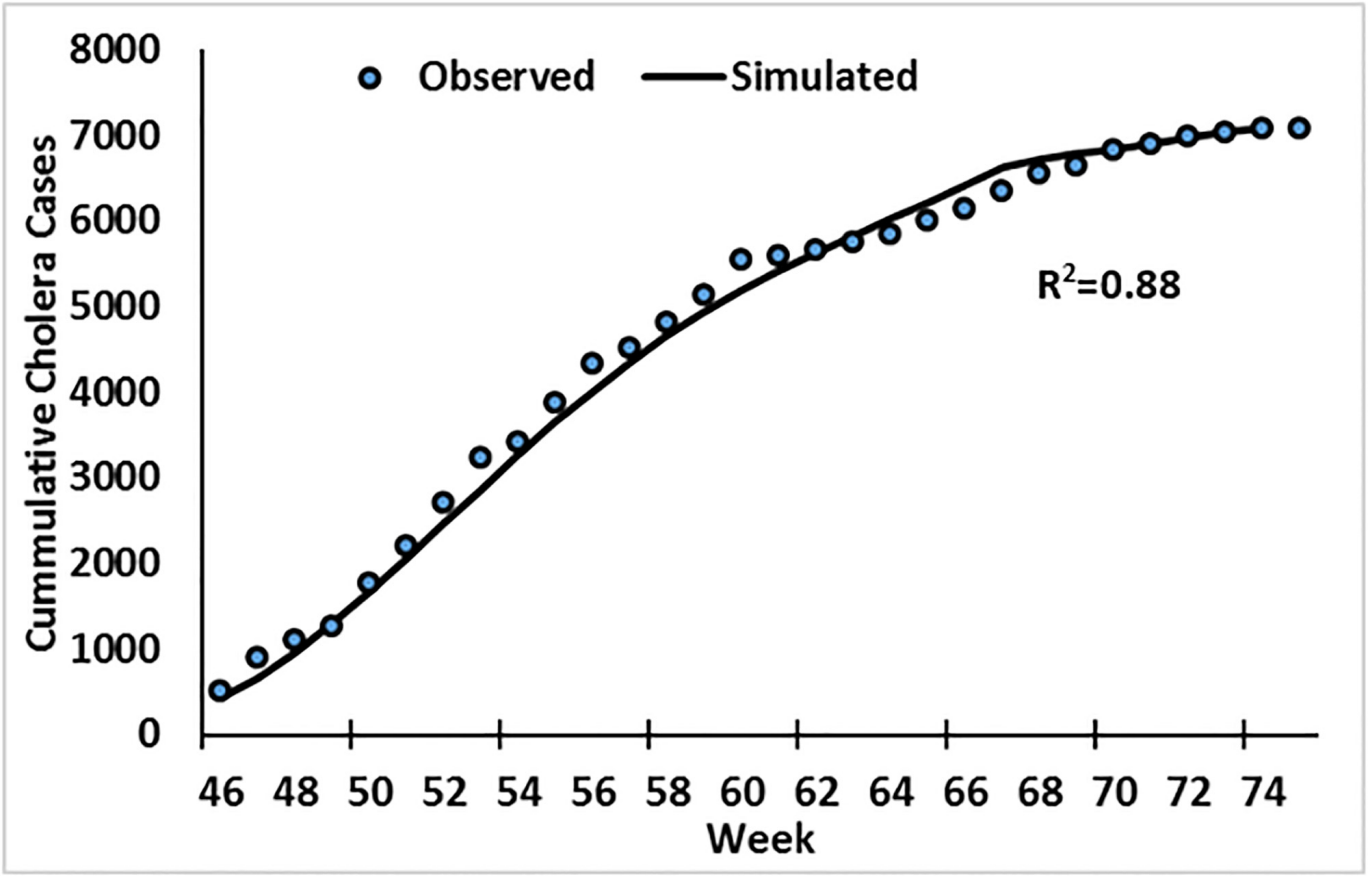
Calibration of the model using Mashonaland East cholera data. (Starting week 46 represent November 13, 2008).

Mathematical models, such as compartmental representation of population [20], are often useful in understanding role of different environmental processes and associated variables that lead to disease outbreak. Therefore, after development of a SEIR model (details in supplementary information), calibration (Fig 8; fitted R^2^ = 0.88) using weekly cholera case data for Mashonaland East, three scenarios were used to determine the role, and possible association, of hydroclimatic processes as a trigger of epidemic cholera.

#### Case 1: Control (equilibrium scenario)

**Fig 9:**
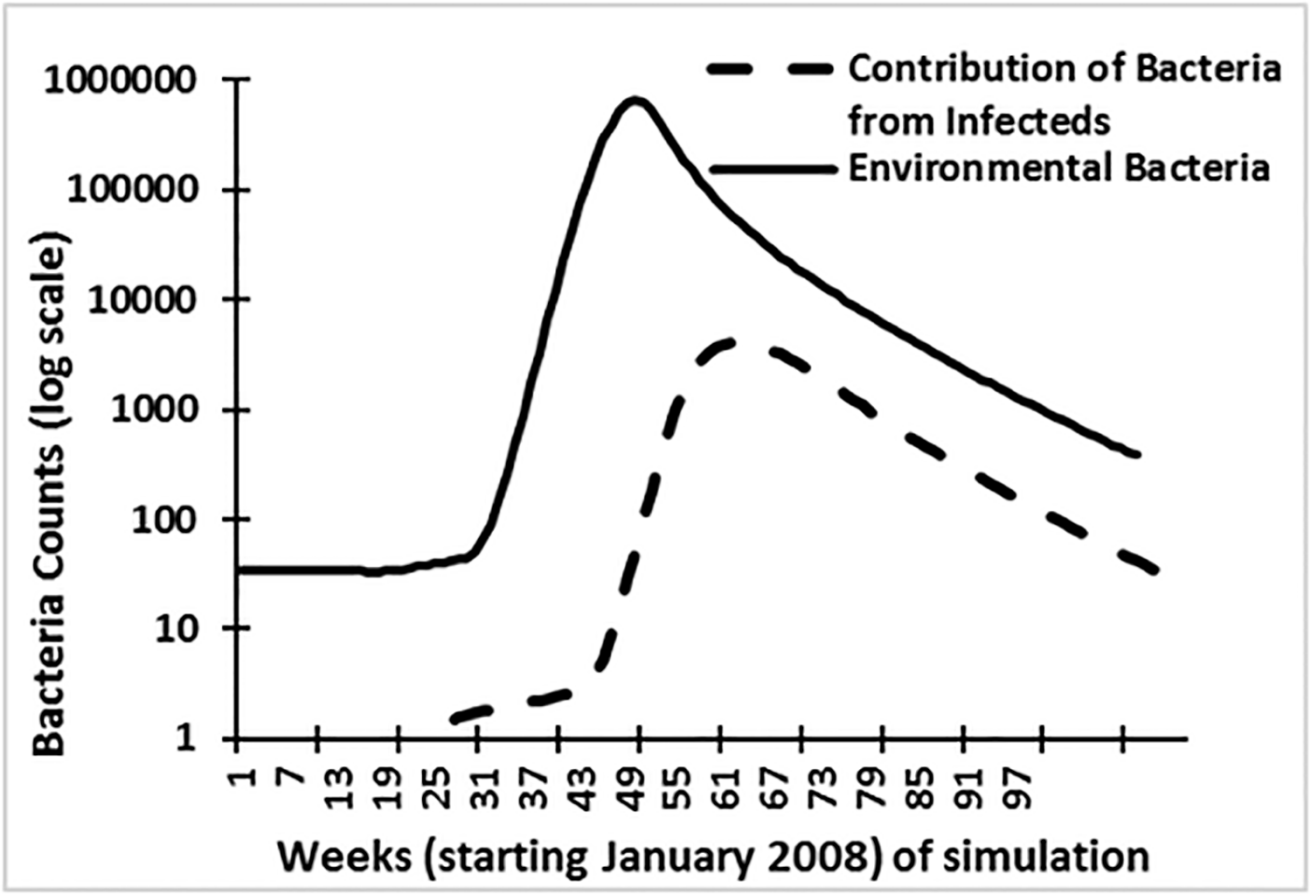
Simulated contribution of bacteria from infected population and growth of environmental bacteria.

The model for this case scenario was calibrated using the assumptions that the susceptible population was greater than 1 and that there were no infected individuals (or carriers) present in the population. If our hypothesis that cholera outbreaks in Zimbabwe are related to hydroclimatic processes is valid, then environmental bacteria in the aquatic medium should have increased in number (presence) at a higher rate than the number of cases occurring via secondary route of infection, primarily shedding of cholera bacteria from infected humans to the environment. Environmental and human contributions to the overall pathogen pool is shown in Fig 9. The data suggest that environmental bacteria numbers peaked at least one month in advance compared to human transmission (secondary transmission).

#### Case 2: No external hydroclimatic forcing with presence of infected individuals

**Fig 10:**
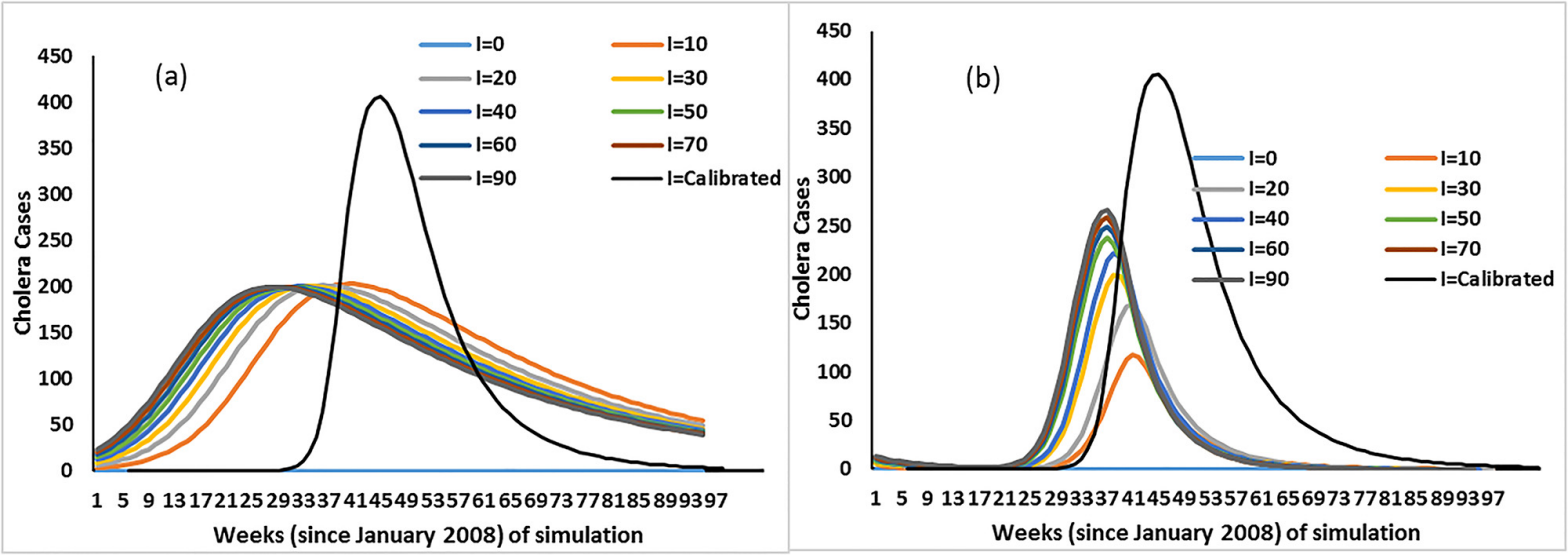
(a) Cholera cases with no external hydroclimatic forcing and environmental bacteria reservoir; (b) Cholera cases with external hydroclimatic forcing but with no environmental bacteria reservoir. (I = 0, 10…90 indicates infected introduced in the population and I = calibrated is the simulated infected cholera cases after calibration of model).

In this scenario, it was assumed that there were no external hydroclimatic forcings and no buildup of cholera bacteria populations in the aquatic system. The assumption was that infected individuals were present and were simulated for each sub-scenario. Fig 10 shows that none of the sub-scenarios was able to replicate the observed infected (I = calibrated) results, indicating that, in absence of appropriate hydroclimatic conditions, a cholera epidemic would not have occurred in the region.

#### Case 3: External hydroclimatic forcing and presence of infected individuals

This scenario is based on the assumption that external hydroclimatic conditions, such as above average temperature followed by above average precipitation, occurred without an enhanced environmental population of cholera bacteria. Similar to case 2 above, sub-scenarios were computed to determine if introduction of infected individuals, with subsequent shedding of cholera vibrios to the aquatic environment, was sufficient to trigger an epidemic. Fig 10 shows that none of the sub-scenarios, until introduction of 90 infected individuals was postulated, simulated conditions of the calibrated model (I = calibrated). However, when 10 to 20 infected individuals were introduced, a small increase in the number of cholera cases was predicted. However, without temperature and precipitation affecting the environmental reservoir, cholera epidemics were not indicated in any of the model simulations, giving credence to synergism between the environmental reservoir and external hydroclimatic forcings.

## Summary and Conclusion

Cholera is likely to occur in epidemic regions, namely those with nearby rivers or water bodies and insufficient safe water and sanitation systems, during times when warm air temperatures are followed by heavy precipitation, conditions that are optimum for growth of the cholera bacteria. Heavy rainfall and subsequent flooding accelerate interaction between a contaminated water source and human exposure. Hydroclimatic predictability of a lead time for occurrence of cholera is possible when temperature and rainfall are higher than the long-term average and conditions are favorable for growth of cholera bacteria in the aquatic environment. Satellite data were successfully used to capture hydroclimatic sensitivities of satellite (TRMM) based precipitation and gridded air temperature data to predict cholera outbreaks atleast one month in advance.

Interest in the extremes of the variables of temperature and rainfall has heightened with climate change. Cholera epidemics are likely to occur with high probability if *hydroclimatological risk* [defined as a combination of large scale geophysical processes and environmental conditions conducive to bacterial growth] and *societal risk* [defined as reduced or lack of availability of safe drinking water and sanitation] occur in a community or region. The challenge in developing a cholera prediction model for epidemic regions is to identify environmental conditions and enabling transmission mechanisms. When a major natural disaster strikes a region (town/province) or civil disorder damages living conditions of a population center, a hydroclimate driven cholera tracking mechanism can be made operational by satellite remote sensing, monitor water and sanitation infrastructure, and the displaced population. Convergence of these factors with environmental conditions optimum for growth of the cholera bacteria and knowledge of transmission pathways, can provide a reliable prediction and early warning of cholera epidemics for public health action. In most affected regions, preventive responses are tied to the timing of the outbreaks. Such responses typically depend on the emergence of the index cases in a vulnerable region, where local health departments, in conjunction with public health personnel on the ground, monitor and evaluate the spread of cases. Once an outbreak in confirmed, measures to avoid or reduce infection—mostly in the form of providing safe water access, mobile sanitation facilities, water and sanitation awareness, medication for the infected (such as antibiotics or intravenous fluids), and oral rehydration therapy (ORT) is mobilized. However, due to the lack of long lead predictions, current warnings and responses typically operate with a lead-time of only a few days since the emergence of the index case. Thus, while these preparations are able to save lives and reduce infections, they become effective only after the outbreaks are well under way and thus the disease burden remains high in many regions.

Although, the timing can be anticipated fairly well in endemic regions, the potential magnitude or the location of the outbreak remain unknown—and prepositioning and maintaining the appropriate level of human and material resources in anticipation of an outbreaks remains a difficult task. Predictions can add significant ‘actionable knowledge’ to preemptive preparations. Especially in epidemic regions, predictions can be valuable by adding the potential timing of outbreaks. Thus, armed with the knowledge of imminent outbreaks with a few weeks to months lead-time, vulnerable population groups (such as children and elders) can be preemptively vaccinated, at-risk areas (such as low level areas prone to flooding, areas with water scarcity and quality issues, areas next to bad drainage or effluent discharges, slums, etc) can be targeted with water and sanitation facilities, awareness, and rules enforcement, and public health groups can be prepositioned with ORT, antibiotics, and vaccines [35].

In addition, coupled with advances at the local scale of diarrheal treatment, innovative solutions such as simple filtration measures during extreme weather conditions, and vaccines as proven by improved survival rates, a prediction-based surveillance mechanism would help monitoring vulnerable regions and strengthen ability of the public health community to halt outbreaks early and reduce the disease burden [36]. The challenge to predict and mitigate outbreaks becomes more challenging, when societal change and factors such as civil unrest and natural disasters occur. However, the risks is compounded with environmental factors conducive to cholera outbreaks (due to temperature increase, heavy precipitation patters, or drought/flood risk and proliferation of the cholera bacteria), and a robust early warning can contribute to mitigation of cholera outbreaks by enhancing the response capacity and resilience of regional institutions.

## Supporting Information

S1 File This file describes various model development and calibration procedures for mechanistic and logistical regression analysis. SEIR model variables and associated symbols (Table A); parameters and values used for model fitting (Table B); and model parameters for logistical regression for Indus Basin (Table C). Hydroclimatic SEIR model design (Fig A).
(DOCX)

## References

[1] Jutla A, Whitcombe E, Hasan N, Haley B, Akanda A, Huq A, et al Environmental Factors Influencing Epidemic Cholera. Am J Trop Med Hyg. 2013;89: 597–607. 10.4269/ajtmh.12-0721 23897993 PMC3771306

[2] Enserink M. Haiti’s Outbreak Is Latest in Cholera’s New Global Assault. Science. 2010;330: 738–739. 10.1126/science.330.6005.738 21051601

[3] Gaffga NH, Tauxe RV, Mintz ED. Cholera: a new homeland in Africa? Am J Trop Med Hyg. 2007;77: 705–713. 17978075

[4] Mendelsohn J, Dawson T. Climate and cholera in KwaZulu-Natal, South Africa: The role of environmental factors and implications for epidemic preparedness. Int J Hyg Environ Health. 2008;211: 156–162. 10.1016/j.ijheh.2006.12.002 17383231

[5] Lobitz B, Beck L, Huq A, Wood B, Fuchs G, Faruque ASG, et al Climate and infectious disease: Use of remote sensing for detection of Vibrio cholerae by indirect measurement. Proc Natl Acad Sci. 2000;97: 1438–1443. 10.1073/pnas.97.4.1438 10677480 PMC26452

[6] Traerup SLM, Ortiz RA, Markandya A. The Costs of Climate Change: A Study of Cholera in Tanzania. Int J Environ Res Public Health. 2011;8: 4386–4405. 10.3390/ijerph8124386 22408580 PMC3290983

[7] Thompson AA, Matamale L, Kharidza SD. Impact of Climate Change on Children’s Health in Limpopo Province, South Africa. Int J Environ Res Public Health. 2012;9: 831–854. 10.3390/ijerph9030831 22690167 PMC3367281

[8] Alexander KA, Carzolio M, Goodin D, Vance E. Climate Change is Likely to Worsen the Public Health Threat of Diarrheal Disease in Botswana. Int J Environ Res Public Health. 2013;10: 1202–1230. 10.3390/ijerph10041202 23531489 PMC3709313

[9] Reyburn R, Kim DR, Emch M, Khatib A, von Seidlein L, Ali M. Climate variability and the outbreaks of cholera in Zanzibar, East Africa: a time series analysis. Am J Trop Med Hyg. 2011;84: 862–869. 10.4269/ajtmh.2011.10-0277 21633020 PMC3110353

[10] Bompangue D, Giraudoux P, Handschumacher P, Piarroux M, Sudre B, Ekwanzala M, et al Lakes as Source of Cholera Outbreaks, Democratic Republic of Congo. Emerg Infect Dis. 2008;14: 798–800. 10.3201/eid1405.071260 18439365 PMC2600234

[11] Bompangue D, Vesenbeckh SM, Giraudoux P, Castro M, Muyembe J-J, Kebela Ilunga B, et al Cholera ante portas—The re-emergence of cholera in Kinshasa after a ten-year hiatus. PLoS Curr. 2012;4: RRN1310 10.1371/currents.RRN1310 22453903 PMC3299488

[12] Rebaudet S, Sudre B, Faucher B, Piarroux R. Environmental determinants of cholera outbreaks in inland Africa: a systematic review of main transmission foci and propagation routes. J Infect Dis. 2013;208 Suppl 1: S46–54. 10.1093/infdis/jit195 24101645

[13] WHO | Cholera in Zimbabwe—update [Internet]. [cited 6 Oct 2014]. Available: http://www.who.int/csr/don/2008_12_26/en/

[14] WHO | Cholera in Zimbabwe—update 3 [Internet]. [cited 6 Oct 2014]. Available: http://www.who.int/csr/don/2009_03_23/en/

[15] Fernandez MAL, Schomaker M, Mason PR, Fesselet JF, Baudot Y, Boulle A, et al Elevation and cholera: an epidemiological spatial analysis of the cholera epidemic in Harare, Zimbabwe, 2008–2009. BMC Public Health. 2012;12: 442 10.1186/1471-2458-12-442 22708576 PMC3483262

[16] Jutla AS, Akanda AS, Islam S. Tracking Cholera in Coastal Regions Using Satellite Observations1. JAWRA J Am Water Resour Assoc. 2010;46: 651–662. 10.1111/j.1752-1688.2010.00448.x 21072249 PMC2975368

[17] Jutla AS, Akanda AS, Griffiths JK, Colwell R, Islam S. Warming Oceans, Phytoplankton, and River Discharge: Implications for Cholera Outbreaks. Am J Trop Med Hyg. 2011;85: 303–308. 10.4269/ajtmh.2011.11-0181 21813852 PMC3144830

[18] Huq A, Sack RB, Nizam A, Longini IM, Nair GB, Ali A, et al Critical factors influencing the occurrence of Vibrio cholerae in the environment of Bangladesh. Appl Environ Microbiol. 2005;71: 4645–4654. 10.1128/AEM.71.8.4645-4654.2005 16085859 PMC1183289

[19] Islam MS, Siddique AK, Salam A, Akram K, Majumdar RN, Zaman K, et al Microbiological investigation of diarrhoea epidemics among Rwandan refugees in Zaire. Trans R Soc Trop Med Hyg. 1995;89: 506 8560524 10.1016/0035-9203(95)90086-1

[20] Mukandavire Z, Liao S, Wang J, Gaff H, Smith DL, Morris JG. Estimating the reproductive numbers for the 2008–2009 cholera outbreaks in Zimbabwe. Proc Natl Acad Sci. 2011;108: 8767–8772. 10.1073/pnas.1019712108 21518855 PMC3102413

[21] Hosmer D, Lemeshow S. Applied Logistic Regression. John Wiley & Sons, Inc; 1989.

[22] Hosmer DW, Hosmer T, Le Cessie S, Lemeshow S. A comparison of goodness-of-fit tests for the logistic regression model. Stat Med. 1997;16: 965–980. 9160492 10.1002/(sici)1097-0258(19970515)16:9<965::aid-sim509>3.0.co;2-o

[23] Turton A. Three Strategic Water Quality Challenges that Decision-Makers Need to Know About and How the CSIR Should Respond [Internet]. Pretoria: Council for Scientific and Indus trial Research; 2008 11 p. 28 Report No.: CSIR/NRE/WR/EXP/2008/0160/A. Available: http://www.environment.co.za/documents/water/KeynoteAddressCSIR2008.pdf

[24] Akanda A, Jutla A, Gute D, Evans T, Islam S. Reinforcing cholera intervention through prediction-aided prevention. Bull World Health Organ. 2012;90: 243–244. 10.2471/BLT.11.092189 22461722 PMC3314217

[25] Akanda AS, Jutla AS, Colwell RR. Global diarrhoea action plan needs integrated climate-based surveillance. Lancet Glob Health. 2014;2: e69–e70. 10.1016/S2214-109X(13)70155-4 25104658

[26] WHO | WHO responds to health crises facing war-wracked South Sudan [Internet]. [cited 16 Oct 2014]. Available: http://www.who.int/features/2014/health-crisis-south-sudan/en/

[27] WHO. Cholera outbreak in Juba, Central Equatoria State [Internet]. 2014 May p. 5. Available: http://who.int/hac/crises/ssd/sitreps/south_sudan_juba_cholera_update_16may2014.pdf?ua=1

[28] Central African Republic: Severe Local Storm—Jun 2011. In: ReliefWeb [Internet]. [cited 16 Oct 2014]. Available: http://reliefweb.int/disaster/st-2011-000073-caf

[29] CDC Foundation. Cholera Outbreak Investigation in the Central African Republic [Internet]. p. 20. Available: http://www.cdcfoundation.org/sites/default/files/upload/pdf/2011CholeraOutbreakReport.pdf

[30] Rwanda: Floods—Apr 2012. In: ReliefWeb [Internet]. [cited 16 Oct 2014]. Available: http://reliefweb.int/disaster/fl-2012-000067-rwa

[31] MIH R. Cholera Outbreak in Kinunu and Biruyi Health Centers: Weekly Epidemiological Bulletin [Internet]. Rwanda Biomedical Centre/Institute of HIV/AIDS,Diseases Prevention & C ontrol, Kigali; 2012 10 p. 5 Available: http://www.rbc.gov.rw/IMG/pdf/epidemiological_bulletin_for_week_42.pdf

[32] 9 FCCOS in C| C of B| W| A water news A, Pm 2010 at 2:32. Flooding Causes Cholera Outbreak Spread in Cameroon. In: Circle of Blue WaterNews [Internet]. [cited 16 Oct 2014]. Available: http://www.circleofblue.org/waternews/2010/world/flooding-causes-cholera-outbreak-spread-in-cameroon/

[33] Cameroon cholera deaths rising as heavy rains bring flooding. In: ReliefWeb [Internet]. 3 Aug 2010 [cited 16 Oct 2014]. Available: http://reliefweb.int/report/cameroon/cameroon-cholera-deaths-rising-heavy-rains-bring-flooding

[34] World Health Organization. Global Task Force on Cholera Control [Internet]. Geneva; p. 2 Available: http://www.who.int/cholera/countries/CameroonCountryProfile2011.pdf

[35] World Health Organization. Global Task Force on Cholera Control: Mozambique [Internet]. Geneva; p. 2 Available: http://www.who.int/cholera/countries/MozambiqueCountryProfile2013.pdf

[36] Relief Web. Southern Africa: Floods Situation Report No. 6 (as of 15 February 2013). In: ReliefWeb [Internet]. 15 Feb 2013 [cited 16 Oct 2014]. Available: http://reliefweb.int/report/mozambique/southern-africa-floods-situation-report-no-6-15-february-2013

