# Supplementary material for: Satellite Based Assessment of Hydroclimatic Conditions Related to Cholera in Zimbabwe
Source: PLoS One. 2015 Sep 29;10(9):e0137828. doi: 10.1371/journal.pone.0137828 (PMC4587952; doi:10.1371/journal.pone.0137828)
Supplement: S1 File — SEIR model variables and associated symbols (Table A); parameters and values used for model fitting (Table B); and model parameters for logistical regression for Indus Basin (Table C). Hydroclimatic SEIR model design (Fig A). (DOCX) [file pone.0137828.s001.docx]

**Hydroclimatic SEIR Model**

Susceptible-Exposed-Infected-Recovered (SEIR) [1] model was developed to understand the climate relationship of cholera in Zimbabwe. The basic model structure is shown in Figure S1 and was composed of six compartments, including the susceptible (S), Exposed (E), Infected (I) and Recovered(R) population and two reservoirs of bacteria-one shed from humans (B_I_) and other environmental driven cholera (B_env_). The interaction between the two reservoirs is governed by a series of differential equations, *i* through *vi*. Various variables and parameters are described in Table S1 and S2 respectively.

$\frac{dS}{dt}=\eta\left( S+E+I \right)+\sigma R-\left[ \left( \mu+\alpha_{WASH}.N_{Exp} \right)S \right]$ i$\frac{dE}{dt}=\alpha_{WASH}.N_{Exp}.S-\left( \mu+\beta_{c}.\frac{B_{Env}}{K+B_{env}} \right)E$ ii$\frac{dI}{dt}=\beta_{C}.\frac{B_{Env}}{K+B_{env}}E-\left( \mu_{I}+\gamma\right)I$ iii$\frac{dR}{dt}=\gamma I-\left( \mu+\sigma\right)R$ iv$\frac{dB_{I}}{dt}=\tau I-\psi B_{I}$ v$\frac{dB_{Env}}{dt}=\psi B_{I}+\left( \omega_{Env}\times Pr\left( {cholera}_{hydroclimate} \right)-\theta\right)B_{Env};$ vi

$Pr\left( {cholera}_{hydroclimate} \right)=\frac{e^{a+bx_{n}}}{1+e^{a+bx_{n}}}$ vii

Cholera SIR models generally start with the premise that the cholera bacteria are transmitted primarily via human to human interaction. Recently, the role of indirect transmission via environmental reservoirs has been introduced in SIR models (e.g., [2]). Studies to date have highlighted the role of environmental conditions in creating a seasonality of cholera [3] but do not elaborate on plausible physical mechanisms that explain the seasonality of cholera . These models capture the dynamics of the disease once the outbreak has been initiated. Most SIR models focus on human to human transmission, assuming exponential decay of the bacteria in their reservoir, despite the fact that phenomenon is not commonly observed [2]. In these models, hydroclimatic conditions are emphasized and the basic reproductive number (defined as the number of secondary cases caused by a small number of infected individuals) is used as a central concept [1]. But, these models cannot create (or replicate) seasonality in the disease dynamics unless some of the model parameters are *a-priori* chosen to vary seasonally. Instead of *a-priori* choosing *transmission* mechanisms (primary or secondary) in an arbitrary choice of parameters , our Hydroclimatic-SEIR model assumes that the population in an epidemic region is exposed to two different transmission routes: one through the environment and the other through humans. Our model structure explicitly recognizes climatic connections as the forcing terms for growth of the environmental bacteria and are further linked with the pathogen shed by infected individuals. One of the challenging aspects of the SEIR model was calculation of the probability of cholera occurrence [Pr(cholera_hydroclimate_)] since the historical time series for epidemic outbreaks was not available for Zimbabwe prior to year 2008. Therefore we assumed the probability of occurrence of cholera being 1 if the air temperature is higher than average and is followed by higher than average precipitation. When a longer time series of epidemic cholera becomes available in the future, this relationship may be calculated based on logistical regression models. The model was developed using a systems thinking approach [4,5] and calibrated in a trial and error methodology, based on our previous studies of model development, calibration, and validation [4,5].

S

I

R

E

B_I_

B_Env_

$$\beta_{C}\frac{B_{Env}}{K+B_{env}}$$

$$\alpha_{WASH}.N_{Exp}$$

$$\gamma$$

μ

μ

μ

η

σ

θ

τ

ψ

$$\omega_{Env}.Pr\left( {cholera}_{hydroclimate} \right)$$

μ_I_

**Figure A :Hydroclimatic SEIR model design**

**Table A: Model variables and associated symbols**

| **Variable** | **Symbol** |
| --- | --- |
| Susceptible population | $S$ |
| Exposed population | $E$ |
| Infected population | $I$ |
| Recovered population | $R$ |
| Reservoir of cholera bacteria from Infected population | $B_{I}$ |
| Environmental reservoir of cholera bacteria | $B_{Env}$ |

**Table B: Parameters and values used for model fitting**

| **Parameter** | **Symbol** | **Value** | **Source** |
| --- | --- | --- | --- |
| **Natural human birth rate** | $\eta$ | (43.5y)^-1^ | [1] |
| **Immunity rates** | $\sigma$ |  | N |
| **Natural human death rate** | $\mu$ | (17.27y)^-1^ | [6] |
| **Combined water and sanitation access** | $\alpha_{WASH}$ | 46% | [7] |
| **Population exposure rate** | $N_{Exp}$ | 10% | [7] |
| **Combined infection rate** | $\beta_{C}$ | 0.03 d^-1^ | F |
| **Death rate of infected population** | $\mu_{I}$ | 0.006 y^-1^ | [1] |
| **Rate of contribution to V. cholera in aquatic environment** | $\tau$ | 10 cells.ml^-1^d^-1^per person | [1] |
| **Rate of recovery from cholera** | $\gamma$ | 5 d^-1^ | [1] |
| **Contribution of infected bacteria (B_I_) to environmental reservoir** | $\psi$ | 1 d^-1^ | F |
| **Growth rate of vibrios in the environment** | $\omega_{Env}$ | 0.05 d^-1^ | F |
| **Death rate of vibrios in the environment** | $\theta$ | (30 d)^-1^ | [1] |
| **Concentration of *V. cholera* in environment (ID_50_)** | $K$ | 10^6^ cells/ml | [1] |
| **Probability of cholera occurrence** | $Pr\left( {cholera}_{hydroclimate} \right)$ | Ranges between 0 and 1 | C |
|  | | | |

F=fitted parameter; C=calculated; N= negligible value (no effect in model)

**Table C: Model parameters for logistical regression for Indus Basin.**

|  | **α** | **β*** | **γ*** |
| --- | --- | --- | --- |
| **Delhi** | -2.283 | 0.0034 | 0.1238 |
| **Lahore** | -8.72 | 0.0914 | 0.0633 |
| **Ludhiana** | -10.36 | 0.0974 | 0.0402 |
| **Sialkot** | -7.44 | 0.0612 | 0.1099 |
| **Rawalpindi** | -7.69 | 0.0811 | 0.0268 |
| **Peshawar** | -7.15 | 0.0712 | 0.048 |
| **Dera Ismail Khan** | -9.21 | 0.0855 | 0.08 |
| **Multan** | -8.73 | 0.0739 | 0.061 |
| **Sirsa** | -3.769 | 0.0261 | 0.0637 |
| * | | | |

p-values <0.05; β (temperature); γ (precipitation); α (constant)

**References**

1. Mukandavire Z, Liao S, Wang J, Gaff H, Smith DL, Morris JG. Estimating the reproductive numbers for the 2008-2009 cholera outbreaks in Zimbabwe. Proc Natl Acad Sci. 2011;108: 8767–8772. doi:10.1073/pnas.1019712108

2. Joh RI, Wang H, Weiss H, Weitz JS. Dynamics of Indirectly Transmitted Infectious Diseases with Immunological Threshold. Bull Math Biol. 2009;71: 845–862. doi:10.1007/s11538-008-9384-4

3. Koelle K, Rodó X, Pascual M, Yunus M, Mostafa G. Refractory periods and climate forcing in cholera dynamics. Nature. 2005;436: 696–700. doi:10.1038/nature03820

4. Elshorbagy A, Jutla A, Barbour L, Kells J. System dynamics approach to assess the sustainability of reclamation of disturbed watersheds. Can J Civ Eng. 2005;32: 144–158. doi:10.1139/l04-112

5. Elshorbagy A, Jutla A, Kells J. Simulation of the hydrological processes on reconstructed watersheds using system dynamics. Hydrol Sci J. 2007;52: 538–562. doi:10.1623/hysj.52.3.538

6. UNDESA. World Population Prospects, the 2012 Revision [Internet]. [cited 29 Sep 2014]. Available: http://esa.un.org/unpd/wpp/index.htm

7. The World Bank. Water Supply and Sanitation in Zimbabwe [Internet]. The World Bank; Available: http://www.wsp.org/sites/wsp.org/files/publications/CSO-Zimbabwe.pdf
